# Abnormal bone mineral density and content in girls with early-onset anorexia nervosa

**DOI:** 10.1186/s40337-020-00365-6

**Published:** 2021-01-10

**Authors:** Julia Clarke, Hugo Peyre, Marianne Alison, Anne Bargiacchi, Coline Stordeur, Priscilla Boizeau, Grégor Mamou, Sophie Guilmin Crépon, Corinne Alberti, Juliane Léger, Richard Delorme

**Affiliations:** 1 Child and Adolescent Psychiatry Department, Assistance Publique-Hôpitaux de Paris, Robert Debré University Hospital, Paris, France; 2 Université de Paris, Institute of Psychiatry and Neuroscience of Paris, INSERM U1266, Paris, France; 3 Université de Paris, INSERM UMR 1141, Paris, France; 4 Radiology Department, Assistance Publique-Hôpitaux de Paris, Robert Debré University Hospital, Pediatric, Paris, France; 5 Unit of Clinical Epidemiology, Assistance Publique-Hôpitaux de Paris, Robert Debré University Hospital, Paris, France; 6 Department of Pediatric Endocrinology and Diabetology & Reference centre for Growth and Development Endocrine diseases, Assistance Publique-Hôpitaux de Paris, Robert Debré University Hospital, Paris, France; 7 INSERM UMR-S 1123 ECEVE and CIC-EC 1426, Paris, France

**Keywords:** Anorexia nervosa, Early onset, Bone mineral density, Bone mineral content, Pubertal maturation

## Abstract

**Background:**

Early-onset anorexia nervosa (EO-AN) represents a significant clinical burden to paediatric and mental health services. The impact of EO-AN on bone mineral abnormalities has not been thoroughly investigated due to inadequate control for pubertal status. In this study, we investigated bone mineral abnormalities in girls with EO-AN regardless of pubertal development stage.

**Method:**

We conducted a cross-sectional study of 67 girls with EO-AN (median age = 12.4 [10.9–13.7 years]) after a median duration of disease of 1.3 [0.6–2.0] years, and 67 healthy age-, sex-, pubertal status- matched control subjects. We compared relevant bone mineral parameters between groups: the total body bone mineral density [TB-BMD], the lumbar spine BMD [LS-BMD], the total body bone mineral content [TB-BMC] and the ratio of the TB-BMC to lean body mass [TB-BMC/LBM].

**Results:**

TB-BMD, TB-BMC, LS-BMD and TB-BMC/LBM were all significantly lower in patients with AN compared to controls. In the EO-AN group, older age, later pubertal stages and higher lean body mass were associated with higher TB-BMC, TB-BMD, and LS-BMD values.

**Discussion:**

Girls with EO-AN displayed deficits in bone mineral content and density after adjustment for pubertal maturation. Age, higher pubertal stage and lean body mass were identified as determinants of bone maturation in the clinical population of patients with EO-AN. Bone health should be promoted in patients, specifically in those with an onset of disorder before 14 years old and with a delayed puberty.

## Plain ENGLISH summary

Early-onset anorexia nervosa (EO-AN) – beginning before the age of 14 years and before menarche – represents a significant clinical burden to paediatric and mental health services, with serious medical, psychiatric and psychosocial consequences. The impact of EO-AN on bone mineral abnormalities has not been thoroughly investigated due to inadequate control for pubertal status. In this study, we investigated bone mineral abnormalities in girls with EO-AN regardless of pubertal development stage. We compared bone mineral parameters in 67 girls with EO-AN and 67 healthy age-, sex-, pubertal status- matched control subjects. In our study, all parameters of bone quality were significantly lower in patients with AN compared to controls, showing that girls with EO-AN displayed deficits in bone quality even though the duration of illness was short in these young patients. Bone health should be promoted in patients, specifically in those with an onset of disorder before 14 years old and with a delayed puberty.

## Background

Anorexia nervosa (AN) is characterised by the persistent restriction of energy intake relative to requirements, leading to significantly low body weight, and an intense fear of gaining weight or becoming fat [1]. AN, which has a prevalence of 1% in the general population, has the highest mortality rate of all psychiatric disorders, and serious medical, psychiatric and psychosocial consequences [2, 3]. In most patients AN begins during adolescence or early adulthood, but recent studies have reported an increase in the incidence of early-onset AN (EO-AN), beginning before the age of 14 years [4] and before menarche [5]. EO-AN is a single entity in the broad spectrum of eating disorders, with a significant and specific clinical burden on paediatric and mental health services [6, 7]. The epidemiological and clinical profile of EO-AN display some differences from AN with an onset during adolescence or early adulthood, such as a different sex ratio less unbalanced in favor of women, greater severity of symptoms and specific neurocognitive features [8, 9].

Low spine and whole-body bone mineral density (BMD) and bone mineral content (BMC) have been reported to be associated with AN [10, 11]. These deficits result in a higher risk of spontaneous compression fractures of the vertebrae [12], even in adolescents [13]. More than 50% of adolescents with AN have a BMD more than one standard deviation lower than the mean for age, and approximately 10% of these adolescents have BMD values more than two standard deviations below the mean [14]. This negative effect of the disease on bone health has been found to persist 5 to 10 years after recovery from a severely low BMI at adolescence [15]. In adolescents and adults with AN, the lowest body mass index (BMI) reached during the illness and the total duration of illness (from early symptoms to remission) have consistently been identified as the main factors underlying low BMD [13, 16]. For example, mean bone density loss has been estimated at 2.5% per year in young women with active AN [16]. However, recent findings suggest that the inappropriately low bone densities of patients with AN are driven more by a deficit of bone metabolism in a critical period of development rather than by bone loss (13, [17]. By the age of 18 years, 95% of peak bone mass is achieved, and trabecular bone density increases by about 20% during pubertal growth [18, 19]. Poor bone accrual and a lower peak bone mass in late childhood and early adolescence were therefore thought to have a significant effect on trabecular bone morphology and skeletal strength [20]. Bone acquisition in late childhood is influenced mostly by pubertal stage and growth-related changes in body and skeleton size (including age, height and weight) [21]. During puberty, both nutritional and hormonal factors play major roles in the process of bone development. Increases in bone mineralisation are related to nutritional status (assessed by measuring weight and height) and growth hormone and sex steroid concentrations [22]. Oestrogens are thought to play a key role in increasing and maintaining bone mass in adolescent girls and boys [23]. In girls, peak bone mass gain generally occurs in late puberty, with BMD values increasing up to Tanner stage 4 [22].

The impact of EO-AN on BMD and BMC has not been fully investigated. Previous studies were conducted in subjects with AN beginning during or after the last stages of puberty, i.e. a heterogeneous mixture of children and adolescents in terms of chronological age at AN onset, with inadequate control for pubertal status. In comparisons of patients with pubertal delay with controls (matched only for chronological age and not for Tanner stage), sampling bias may artificially increase the estimated impact of AN on BMC and BMD. We hypothesise that undernutrition has an impact in EO-AN regardless of pubertal development stage (early or late puberty), and that this effect may be greater in patients with severe pubertal delay. In this study, we aimed to fill some of these gaps in our knowledge by assessing the difference in dual-energy X-ray absorptiometry parameters between a unique cohort of girls with restrictive AN beginning before the age of 14 years and a healthy control group matched on the basis of gender, chronological age, but also pubertal status.

## Methods

### Populations

This study included all consecutive female patients with AN hospitalised over a period of 5 years from 2010 to 2015 at the Child and Adolescent Psychiatry Department of Robert Debré Paediatric Hospital (Paris, France). Patients were enrolled at the time of DXA acquisition, during hospitalisation in a dedicated unit for the treatment of eating disorders. Girls with a retrospective diagnosis of EO-AN were included in the study. The criteria for EO-AN diagnosis were: restrictive AN based on the DSM 5 (we retroactively reassigned the DSM-5 diagnoses for participants recruited before 2013) [24], with an age at onset of the disorder of less than 14 years and onset before menarche. Both pre-14 and pre-menarche age requirements were met. All patients were of European descent. At the time of data acquisition, pubertal development and amenorrhoea status were rated by experienced paediatric endocrinologists (all patients had a diagnosis of EO-AN, and they were at various stages of pubertal development at the time of the inclusion in the study). The Tanner staging was the Tanner of breast development. The final diagnosis of AN was reached by summing the information from the Schedule for Affective Disorders and Schizophrenia for School-Age Children, Present and Lifetime Version (K-SADS-PL) [25] and data from clinical reports from experts in the field. We also used the K-SADS-PL to screen for comorbid psychiatric conditions.

The control individuals for this study were from a large group of healthy female children and adolescents from the general population (*n*=319). They were aged 6–20 years and were also all of European descent. These individuals were enrolled in a study aiming to establish control values for bone mineral content and body composition [26]. At inclusion, they had no lifetime history of any chronic medical disorder, impaired growth or axis I psychiatric disorders (investigated by a questionnaire completed by the parents) and none of the controls was under any drug treatment at the time of study. Children with height, weight or body mass values more than two standard deviations on either side of the mean were excluded from the control group. None of the study participants was using hormonal contraception. Given the developmental variability of bone mineral content and body composition, all participants with EO-AN from our study were matched with control individuals for Tanner stage. We split Tanner stages into two distinct subgroups, one corresponding to Tanner stage 1 or 2 (early pubertal stages) and the other to Tanner stages 3 to 5 (late stages of puberty), to prevent a lack of power and the biases introduced by multiple testing.

Height and weight were expressed as standard deviation scores (SDS) for age and sex [27]. We also calculated body mass index (BMI) (kg/m^2^) as a SDS for age and sex [28]. Pubertal development was assessed according to Tanner stage [29].

### Ethics statement

The local ethics committee approved the protocol (Comité de l’Evaluation des Projets de Recherche Biomédicale de Robert Debré).

### Dual-energy X-ray absorptiometry

We estimated the BMC and BMD (corresponding to BMC divided by total bone area) for the total body (TB) and for the lumbar spine (LS), L2–L4, by dual-energy X-ray absorptiometry (DXA, GE Lunar Prodigy Corp., Madison. WI). Bone mineral measurements were expressed as standard deviation scores (SDS) for chronological age. TB DXA provided an estimate of body composition: lean body mass (LBM) and body fat mass (BFM), expressed as a percentage. Given the complex relationship between BMC, LBM, age and growth during puberty, we also calculated the TB-BMC/LBM ratio [30, 31].

### Statistical analysis

The clinical characteristics of patients with AN and control individuals were compared in appropriate tests. Comparisons of quantitative measures between the patient and control individual groups are made using type III chronological age-adjusted tests from mixed models with a random effect on the pair. We compared relevant bone mineral (TB-BMD and LS-BMD, TB-BMC, TB-BMC/LBM) and body composition (BFM and LBM) measurements between individuals with EO-AN and controls, with adjustment for chronological age. Sensitivity analyses were also conducted separately on subjects in the early (Tanner stages 1 or 2) and late (Tanner stages 3 to 5) stages of puberty. Holms correction for multiple testing was applied to control for type 1 error. The *p*-values indicated in the tables are the corrected *p*-values. We used a linear regression model to identify the clinical characteristics (chronological age (years)), BMI (SDS), Tanner status (early vs. later stages), duration of AN, fat mass (kg) and lean mass (kg) associated with our four bone mineral parameters (TB-BMD and LS-BMD, TB-BMC, TB-BMC/LBM). All tests were two-tailed. Statistical analyses were performed with SAS 8.2 software (SAS Institute Inc., Cary, North Carolina).

## Results

### Clinical characteristics

**Fig. 1:**
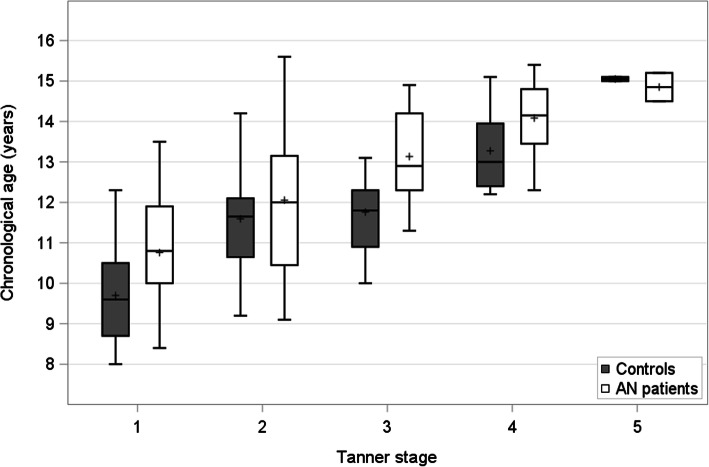
Box plots of Tanner stages in patients vs. controls individuals (*n*=134) and distribution by age

We enrolled 134 girls in the study: 67 patients with EO-AN and 67 female control children and adolescents matched for Tanner stage **(**Table 1**)**. At inclusion, the median duration of the illness was 1.3 (0.6; 2.0) years, 19% of the patients (*n*=13) had secondary amenorrhoea, and 57% (*n*=38) were at Tanner stage 1 or 2. As expected, current BMI was lower for the patients with EO-AN than for the controls. For a given Tanner stage, patients with AN were older than the controls, consistent with the widely reported puberty delay in patients with EO-AN **(**Fig. 1).

**Table 1:** Clinical characteristics of the study population

	EO-AN patients	Control individuals	Patients vs. control individuals
	*N*=67	*N*=67	
	median (25th percentile;75th percentile)	median (25th percentile;75th percentile)	*p*-value
Chronological age (yrs)	12.4 (10.9; 13.7)	11.6 (9.9; 12.4)	*p*< 0.01
Tanner pubertal stage			
1 and 2	38 (57%)	38 (57%)	
3 to 5	29 (43%)	29 (43%)	
Height (SDS)	−0.13 (− 0.86; 1.03)	0.76 (− 0.21; 1.45)	*p*< 0.01
Weight (SDS)	−1.46 (−2.01; − 0.83)	0.80 (0.22; 1.45)	*p*< 0.01
BMI (kg/m^2^)	14.0 (12.7; 15.2)	18.1 (16.5; 20.1)	*p*< 0.01
BMI (SDS)	−2.20 (−3.25; − 1.24)	0.53 (− 0.29; 1.16)	*p*< 0.01
Secondary amenorrhoea	13 (19%)		
Age at EO-AN onset (yrs)	11.0 (9.5; 12.5)		
Disease duration (yrs)	1.3 (0.6; 2.0)		

In bold: *p*-value < 0.01

*BMI* Body Mass Index, *EO-AN* Early Onset Anorexia Nervosa

### Bone mineral content and body composition

**Fig. 2:**
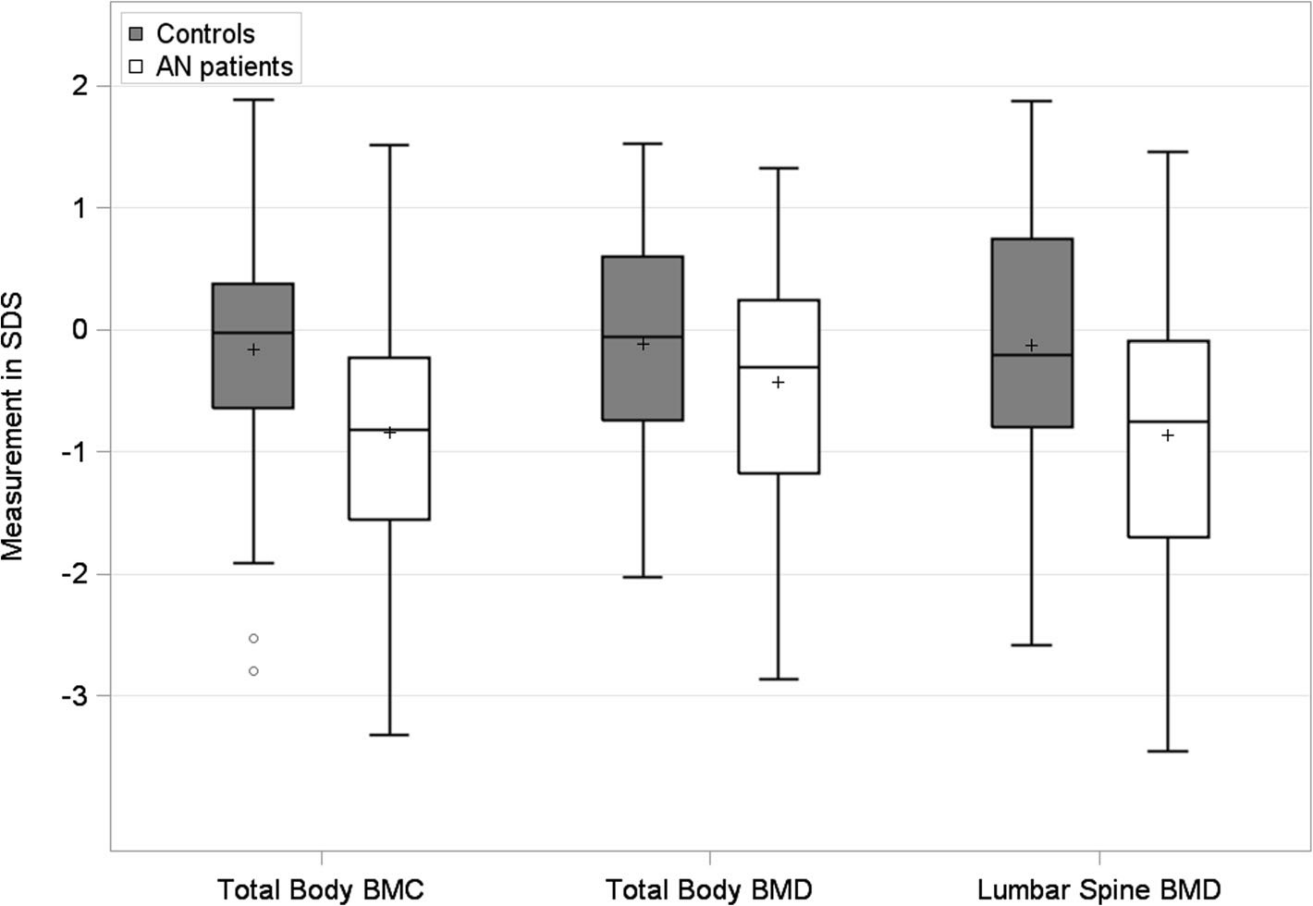
Box plots of total body bone mineral content (BMC), total body bone mineral density (BMD) and lumbar spine BMD in patients vs. controls (*n*=134)

As expected, patients with EO-AN had a lower BFM and a higher LBM, expressed as percentages, than the controls (*p*< 0.01). As shown Table 2, TB-BMD (*p*< 0.01), TB-BMC (*p*< 0.01), LS-BMD (*p*< 0.01) and TB-BMC/LBM (*p*=0.02) were all significantly lower in patients with AN compared to control girls **(**Table 2**,** Fig. 2).

**Table 2:** Comparison of bone mineral characteristics and body composition in 134 children: 67 with EO-AN vs 67 Tanner stage matched control individuals, adjusted for chronological age

	*EO-AN patients*	*Control individuals*	*Patients* vs. *control individuals*
	*N=67*	*N=67*	
	*median (25th percentile;75th percentile)*	*median (25th percentile;75th percentile)*	*p-value*
BFM (% total weight)	10.9 (6.6; 16.2)	23.0 (18.0; 27.3)	< 0.01
LBM (% total weight)	84.9 (78.1; 89.2)	71.3 (67.0; 76.3)	< 0.01
TB LBM/height	171 (156; 187)	187 (169; 210)	< 0.01
TB BMC (SDS)	−0.82 (−1.55; − 0.23)	− 0.02 (− 0.64; 0.38)	< 0.01
TB BMC/LBM	0.21 (− 0.53; 1.04)	−0.32 (− 0.78; 0.46)	0.02
TB BMD (SDS)	− 0.30 (− 1.17; 0.24)	−0.06 (− 0.74; 0.60)	< 0.01
LS BMD (SDS)	− 0.75 (− 1.70; − 0.09)	− 0.20 (− 0.79; 0.75)	< 0.01

*BFM* Body Fat Mass, *EO-AN* Early Onset Anorexia Nervosa, *LBM* Lean Body Mass, *TB-BMC* Total Body Bone Mineral Content, *TB-BMC/LBM (SDS)* Ratio of the TB-BMC to Lean Body Mass (LBM), *TB-BMD (SDS)* Total Body Bone Mineral Density, *LS-BMD (SDS)* Lumbar Spine Bone Mineral Density

### Predictors of bone parameters in EO-AN patients

In our generalised linear regression model higher TB-BMC, TB-BMC/LBM, TB-BMD, and LS-BMD values were associated with a higher chronological age at the time of inclusion (*p*< 0.001 in all 4 models), regardless of body mass index. We also found that higher pubertal stage (Tanner 1 or 2 versus 3, 4 or 5; *p*=0.004 for TB-BMC, *p*=0.02 for TB-BMD and *p*=0.001 for LS-BMD) and higher lean body mass (*p*< 0.001 in all 4 models) were positively associated with higher TB-BMC, TB-BMD, and LS-BMD (*p*< 0.001 in all 4 models), regardless of body mass index and age **(**Table 3**).** Among EO-AN patients, girls at later pubertal stages had higher TB-BMC, TB-BMD, and LS-BMD values. Age, later pubertal stage and higher lean body mass were determinants of total body and lumbar spine bone mineral content and bone mineral density in the clinical population of EO-AN patients.

**Table 3:** Linear regression model of clinical characteristics (chronological age, BMI, Tanner status, duration of AN, amenorrhea, body fat mass and lean body mass) associated with the four bone mineral parameters (TB-BMD and LS-BMD, TB-BMC, TB-BMC/LBM)

		*TB BMC (SDS)*			*TB BMC/LBM (%)*			*TB BMD (SDS)*			*LS BMD (SDS)*	
		*N=67*			*N=67*			*N=67*			*N=67*	
	β	*IC-95%*	*p-value*	β	*IC-95%*	*p-value*	β	*IC-95%*	*p-value*	β	*IC-95%*	*p-value*
Chronological age (yrs)	166.2	[133.92; 198.49]	< 0.01	0.13	[0.06; 0.20]	< 0.01	0.03	[0.02; 0.04]	< 0.01	0.06	[0.04; 0.07]	< 0.01
Pubertal stage			< 0.01			0.55			0.02			< 0.01
1 and 2	*ref.*	*ref.*		*ref.*	*ref.*		*ref.*	*ref.*		*ref.*	*ref.*	
3 to 5	217.25	[71.32; 363.17]		0.10	[−0.24; 0.44]		0.04	[0.01; 0.08]		0.09	[0.04; 0.15]	
BMI (SDS)	15.45	[−33.00; 63.90]	0.53	−0.13	[− 0.24; − 0.02]	0.07	0.01	[0.00; 0.02]	0.16	0.02	[0.00; 0.04]	0.02
FBM (kg)	−25.10	[−66.67; 16.47]	0.23	0.02	[−0.07; 0.11]	0.61	0.01	[−0.01; 0.00]	0.32	−0.01	[− 0.02; 0.01]	0.45
LBM (kg)	65.11	[53.86; 76.35]	< 0.01	0.04	[−0.01; 0.08]	0.10	0.01	[0.01; 0.01]	< 0.01	0.02	[0.01; 0.02]	< 0.01
Disease duration (yrs)	−60.79	[− 129.85; 8.27]	0.08	−0.03	[− 0.19; 0.12]	0.67	− 0.01	[− 0.02; 0.01]	0.31	−0.03	[− 0.05; 0.00]	0.06

*BMI* Body mass index, *BFM* Body Fat Mass, *LBM* Lean Body Mass, *TB-BMC* Total Body Bone Mineral Content, *TB-BMC/LBM (SDS)* Ratio of the TB-BMC to Lean Body Mass (LBM), *TB-BMD (SDS)* Total Body Bone Mineral Density, *LS-BMD (SDS)* Lumbar Spine Bone Mineral Density

## Discussion

This observational study provided extensive data for the assessment of bone mineral density and content in a well-characterised cohort of girls with severe restrictive AN beginning before the age of 14 years. Our findings highlight the abnormal bone mineral composition of these patients, affecting bone mineral content and, to a lesser extent, bone mineral density. Moreover, lumbar spine density was more affected than total body density. The findings of our study are consistent with those of previous studies. Previous studies on prepubertal children and children in the *early stages of puberty* have reported that bone mineral content is a more accurate measurement of bone acquisition than bone mineral density [21]. Moreover, the trabecular bone (lumbar spine) has been shown to be more severely affected than cortical bone in girls with EO-AN [8, 9]. Women with AN beginning during adolescence are known to have a lower bone density than women developing this condition during adulthood, despite similar durations of amenorrhoea [32]. In our study, the decrease in bone mineral content and spinal bone mineral density were very significant (about − 0.8 SDS) despite the short duration of disease. Age, later pubertal stage and higher lean body mass were determinants of bone mineral density and content in our study. Duration of illness has been previously shown to be a negative predictor of LS BMD [13]. The duration of illness was not related with BMD and BMC probably due to the narrow range of the duration of the illness in our cohort (median of 1.3 years).

Adolescence is a time window during which bone accrual must be maximal to attain an optimal peak bone mass. Any deficits occurring during this period may be permanent [17, 33]. EO-AN is a severe disease that affects bone mineralisation before puberty and is associated with a high risk of altered bone mineralisation if symptoms persist during puberty. EO-AN had an impact regardless of pubertal development stage, and this effect was strongest in those with the most severe pubertal delay (suffering from both oestrogen deficiency and undernutrition). An inadequate skeletal mass results in fragile bones and a higher risk of fractures, both in childhood and later in life, as adults [34, 35]. Early diagnosis and intervention are required to prevent such complications in this specific population.

One of the strengths of this study is that the pubertal delay in patients with early AN was considered by matching patients and controls for pubertal stage rather than chronological age. Indeed, the hormonal changes occurring during puberty are known to play a crucial role in bone mineralisation [22, 23]. We found differences in bone mineral parameters between EO-AN patients and control individuals at both early (Tanner 1 or 2) and late (Tanner 3, 4 or 5) stages of puberty. Furthermore, pubertal delay was associated with a greater deficit in bone maturation. In populations of adults with chronic AN, it has been suggested that hormonal adaptations to nutrient deficiency (including hypogonadotropic hypogonadism and growth hormone resistance) mediate a loss of bone mass [17, 36]. However, our study set-up was not appropriate for the testing of this hypothesis. Our results must be interpreted in the light of several limitations. First, AN was particularly severe in our sample of patients, who required hospitalisation in a highly specialised eating disorders unit, with the nationwide recruitment of patients particularly resistant to ambulatory care. Our sample may not, therefore, be entirely representative of the population of girls with EO-AN (with an onset before the age of 14 and primary amenorrhea). Second, although our sample is the largest sample from this clinical population ever studied, it nevertheless remains small. Our study may therefore lack power (i) to detect subtle differences in bone mineral composition (type 2 error) and (ii) to identify some of the clinical characteristics associated with bone mineral content and density in girls with AN. Finally, it was not possible to use our data to further assess the contribution of biological mechanisms to bone deficits in anorexia nervosa. We did not assess calcium intake or physical activity, although both these factors are known to be major determinants of bone mineralisation. Further studies are required to increase our understanding of the factors affecting bone mineral content and density before the completion of pubertal development in women with early-onset AN.

## Conclusions

Even after a short duration of disease, girls with early-onset anorexia nervosa display bone loss, with severe effects on bone mineral content and lumbar spine density. These results highlight the need for careful and appropriate monitoring of bone mineral characteristics in these patients and the importance of nutrition rehabilitation to allow pubertal development to occur, given the importance of this process for bone mineral acquisition. Girls with EO-AN may have a high risk of fracture throughout life. Bone health should, therefore, be promoted through nutritional and other interventions (e.g. avoidance of toxic substances, physical activity under medical supervision etc.). Future studies should compare prospective bone mineral content and density in young adults with EO-AN and those with later onset of the disease, taking into account the duration of undernutrition, the pubertal delay (including age at menarche and catch-up growth), and the clinical characteristics of AN (recovery versus relapse and chronicity).

## Data Availability

The datasets used and/or analysed during the current study are available from the corresponding author on reasonable request.

## Abbreviations

BFM: Body Fat Mass
BMI: Body Mass Index
DXA: Dual-Energy X-ray Absorptiometry
EO-AN: Early-Onset Anorexia Nervosa
LBM: Lean Body Mass
LS-BMD: Lumbar Spine Bone Mineral Density
SDS: Standard Deviation Scores
TB-BMC/LBM: Ratio of the Total Body Bone Mineral Content to Lean Body Mass
TB-BMC: Total Body Bone Mineral Content
TB-BMD: Total Body Bone Mineral Density

## References

[1] American Psychiatric Association. Diagnostic and Statistical Manual of Mental Disorders: American Psychiatric Association; 2013. [cité 1 mai 2015]. 1. (DSM Library). Disponible sur: http://dsm.psychiatryonline.org/doi/book/10.1176/appi.books.9780890425596.

[2] Arcelus J, Mitchell AJ, Wales J, Nielsen S (2011). Mortality rates in patients with anorexia nervosa and other eating disorders. A meta-analysis of 36 studies. Arch Gen Psychiatry.

[3] Excess mortality, causes of death and prognostic factors in anorexia nervosa. - PubMed - NCBI [Internet]. [cité 14 nov 2018]. Disponible sur: https://www.ncbi.nlm.nih.gov/pubmed/19118319.

[4] Lask B, Bryant-Waugh R (1993). Eating disorders. Br J Hosp Med.

[5] Russell GF (1985). Premenarchal anorexia nervosa and its sequelae. J Psychiatr Res.

[6] Nicholls DE, Lynn R, Viner RM (2011). Childhood eating disorders: British national surveillance study. Br J Psychiatry J Ment Sci.

[7] Pinhas L, Morris A, Crosby RD, Katzman DK (2011). Incidence and age-specific presentation of restrictive eating disorders in children: a Canadian Paediatric Surveillance Program study. Arch Pediatr Adolesc Med.

[8] Mouren-Simeoni MC, Bouvard MP. Anorexie mentale chez l’enfant pré-pubère: particularités cliniques et évolutives. In: Neuropsychiatrie de l’enfance et de l’adolescence: Elsevier; 1993. p. 291–5. [cité 29 avr 2015]. Disponible sur: http://cat.inist.fr/?aModele=afficheN&cpsidt=4815691.

[9] van Noort BM, Lohmar SK, Pfeiffer E, Lehmkuhl U, Winter SM, Kappel V (2018). Clinical characteristics of early onset anorexia nervosa. Eur Eat Disord Rev J Eat Disord Assoc.

[10] Robinson L, Aldridge V, Clark EM, Misra M, Micali N (2016). A systematic review and meta-analysis of the association between eating disorders and bone density. Osteoporos Int J Establ Result Coop Eur Found Osteoporos Natl Osteoporos Found USA.

[11] Schorr M, Thomas JJ, Eddy KT, Dichtel LE, Lawson EA (2017). Meenaghan E, et al. Bone density, body composition, and psychopathology of anorexia nervosa spectrum disorders in DSM-IV vs DSM-5. Int J Eat Disord.

[12] Lucas AR, Melton LJ, Crowson CS, O’Fallon WM (1999). Long-term fracture risk among women with anorexia nervosa: a population-based cohort study. Mayo Clin Proc.

[13] Shepherd S, Kyriakou A, Shaikh MG, McDevitt H, Oakley C, Thrower M (2018). Longitudinal changes in bone parameters in young girls with anorexia nervosa. Bone.

[14] Misra M, Aggarwal A, Miller KK, Almazan C, Worley M, Soyka LA (2004). Effects of anorexia nervosa on clinical, hematologic, biochemical, and bone density parameters in community-dwelling adolescent girls. Pediatrics.

[15] Mumford J, Kohn M, Briody J, Miskovic-Wheatley J, Madden S, Clarke S (2019). Long-term Outcomes of Adolescent Anorexia Nervosa on Bone. J Adolesc Health Off Publ Soc Adolesc Med.

[16] Miller KK, Lee EE, Lawson EA, Misra M, Minihan J, Grinspoon SK (2006). Determinants of skeletal loss and recovery in anorexia nervosa. J Clin Endocrinol Metab.

[17] Fazeli PK, Klibanski A (2018). Effects of Anorexia Nervosa on Bone Metabolism. Endocr Rev.

[18] ADG B-J, Faulkner RA, Forwood MR, Mirwald RL, Bailey DA (2011). Bone mineral accrual from 8 to 30 years of age: an estimation of peak bone mass. J Bone Miner Res Off J Am Soc Bone Miner Res.

[19] Gabel L, Macdonald HM, HA MK (2016). Reply to: Challenges in the Acquisition and Analysis of Bone Microstructure During Growth. J Bone Miner Res Off J Am Soc Bone Miner Res.

[20] Mitchell DM, Caksa S, Yuan A, Bouxsein ML, Misra M, SAM BB (2018). Trabecular Bone Morphology Correlates With Skeletal Maturity and Body Composition in Healthy Adolescent Girls. J Clin Endocrinol Metab.

[21] TAL W, Liu X, Pitukcheewanont P, Gilsanz V (2005). Bone acquisition in healthy children and adolescents: comparisons of dual-energy x-ray absorptiometry and computed tomography measures. J Clin Endocrinol Metab.

[22] Yilmaz D, Ersoy B, Bilgin E, Gümüşer G, Onur E, Pinar ED (2005). Bone mineral density in girls and boys at different pubertal stages: relation with gonadal steroids, bone formation markers, and growth parameters. J Bone Miner Metab.

[23] Singh D, Sanyal S, Chattopadhyay N. The role of estrogen in bone growth and formation: changes at puberty. Cell Health and Cytoskeleton. 2011;3:1–12.

[24] American Psychiatric Association. Diagnostic and statistical manual of mental disorders. 5th ed. Washington: American Psychiatric Association; 2013.

[25] Kaufman J, Birmaher B, Brent D, Rao U, Flynn C, Moreci P (1997). Schedule for Affective Disorders and Schizophrenia for School-Age Children-Present and Lifetime Version (K-SADS-PL): initial reliability and validity data. J Am Acad Child Adolesc Psychiatry.

[26] Léger J, Marinovic D, Alberti C, Dorgeret S, Chevenne D, Marchal CL (2006). Lower bone mineral content in children with type 1 diabetes mellitus is linked to female sex, low insulin-like growth factor type I levels, and high insulin requirement. J Clin Endocrinol Metab.

[27] Sempé (M) (1980). Auxologie, méthode et séquences. Bull Mém Société Anthropol Paris.

[28] Rolland-Cachera MF, Cole TJ, Sempé M, Tichet J, Rossignol C, Charraud A (1991). Body Mass Index variations: centiles from birth to 87 years. Eur J Clin Nutr.

[29] Tanner JM, Whitehouse RH (1976). Clinical longitudinal standards for height, weight, height velocity, weight velocity, and stages of puberty. Arch Dis Child.

[30] Courteix D, Lespessailles E, Loiseau-Peres S, Obert P, Ferry B, Benhamou CL (1998). Lean tissue mass is a better predictor of bone mineral content and density than body weight in prepubertal girls. Rev Rhum Engl Ed.

[31] Högler W, Briody J, Woodhead HJ, Chan A, Cowell CT (2003). Importance of lean mass in the interpretation of total body densitometry in children and adolescents. J Pediatr.

[32] Biller BM, Saxe V, Herzog DB, Rosenthal DI, Holzman S, Klibanski A (1989). Mechanisms of osteoporosis in adult and adolescent women with anorexia nervosa. J Clin Endocrinol Metab.

[33] Misra M, Klibanski A (2014). Anorexia nervosa and bone. J Endocrinol.

[34] Drabkin A, Rothman MS, Wassenaar E, Mascolo M, Mehler PS (2017). Assessment and clinical management of bone disease in adults with eating disorders: a review. J Eat Disord.

[35] Faje AT, Fazeli PK, Miller KK, Katzman DK, Ebrahimi S, Lee H (2014). Fracture risk and areal bone mineral density in adolescent females with anorexia nervosa. Int J Eat Disord.

[36] Schorr M, Miller KK (2017). The endocrine manifestations of anorexia nervosa: mechanisms and management. Nat Rev Endocrinol.

